# Vinaxanthone inhibits Semaphorin3A induced axonal growth cone collapse in embryonic neurons but fails to block its growth promoting effects on adult neurons

**DOI:** 10.1038/s41598-021-92375-w

**Published:** 2021-06-21

**Authors:** Evguenia Ivakhnitskaia, Matthew R. Chin, Dionicio Siegel, Victor H. Guaiquil

**Affiliations:** 1 Department of Ophthalmology and Visual Sciences, University of Illinois-Chicago, Chicago, IL USA; 2 Skaggs School of Pharmacy and Pharmaceutical Sciences, University of California, San Diego, La Jolla, CA USA

**Keywords:** Cell biology, Neuroscience

## Abstract

Semaphorin3A is considered a classical repellent molecule for developing neurons and a potent inhibitor of regeneration after nervous system trauma. Vinaxanthone and other Sema3A inhibitors are currently being tested as possible therapeutics to promote nervous system regeneration from injury. Our previous study on Sema3A demonstrated a switch in Sema3A’s function toward induction of nerve regeneration in adult murine corneas and in culture of adult peripheral neurons. The aim of the current study is to determine the direct effects of Vinaxanthone on the Sema3A induced adult neuronal growth. We first demonstrate that Vinaxanthone maintains its anti-Sema3A activity in embryonic dorsal root ganglia neurons by inhibiting Sema3A-induced growth cone collapse. However, at concentrations approximating its IC50 Vinaxanthone treatment does not significantly inhibit neurite formation of adult peripheral neurons induced by Sema3A treatment. Furthermore, Vinaxanthone has off target effects when used at concentrations above its IC50, and inhibits neurite growth of adult neurons treated with either Sema3A or NGF. Our results suggest that Vinaxanthone’s pro-regenerative effects seen in multiple in vivo models of neuronal injury in adult animals need further investigation due to the pleiotropic effect of Sema3A on various non-neuronal cell types and the possible effect of Vinaxanthone on other neuroregenerative signals.

## Introduction

Semaphorins are a glycoprotein family of which five classes appear in vertebrates and most commonly bind plexin and neuropilin receptors. Although this family of molecules is best known in the context of neural development, Semaphorins are widely expressed and affect angiogenesis and vasculogenesis, cardiac development, bone remodeling, immune cell interactions and migration^1–5^. During embryonic neural development Sema3A is considered a canonical repulsive growth cue; it is well known to cause axonal growth cone collapse and to repel growing axons, processes that are necessary to establish proper patterning in midline crossing neurons in the spinal cord, cortex, and the optic tract^6,7^. Sema3A is thought to act through NRP-1/Plexin A1 receptor complex, although in some cell types Plexin A3 plays a major role in its function^8–10^. The mechanism of Sema3A’s action has not been fully elucidated; however, it is known that interaction of Sema3A with its receptor complex leads to receptor internalization and the initiation of intracellular signaling cascades involving Rho family GTPases and classical kinase pathways (MAP/ERK, PI3K/Akt) that affect actin and, likely, microtubule dynamics within the neuronal growth cone^6,8,11^.

Although Sema3A is widely accepted as chemorepellent and growth-inhibitory during embryonic neuronal development, its role in the adult nervous system is more controversial. Our previous study determined that Sema3A is quickly and robustly upregulated in the injured cornea and switches its function in adult versus embryonic nerves^12^. Contrary to its inhibitory effects on embryonic neurons, we found that Sema3A is as effective as NGF in promoting neuronal elongation and branching in isolated adult PNS neurons and expedites nerve regeneration in vivo in mice that have undergone corneal subbasal nerve injury. Other groups have also found that supplementation with related Semaphorin proteins after corneal injury leads to improvement in epithelial and neuronal healing, including Sema3C and Sema 7A^13,14^.

Multiple other studies have arrived at the opposite interpretation of Sema3A’s effect on nerve regeneration in adult animals. In a landmark study on adult rabbits, expression of Semaphorin III in corneal epithelium resulted in repulsion of corneal nerves both in the uninjured cornea and in corneas that had undergone lamellar keratectomy^15^. Other studies have tested the effect on nerve regeneration by pharmacologic inhibitors of Sema3A, like the *Penicillium* sp. fungus derived metabolites SM-345431 (Vinaxanthone) and SM-216289 (Xanthofulvin)^16–18^. The proposed mechanism of inhibition by Xanthofulvin is due to its direct interaction with Sema3A, preventing Sema3A binding to the NRP-1 receptor^19^. Vinaxanthone is structurally similar to Xanthofulvin and is also thought to block the interaction of Sema3A with its receptor^20^. In vivo studies have shown that addition of Xanthofulvin after rat spinal cord transection promoted neural regeneration^21^, and within the eye, Vinaxanthone was determined to be beneficial for nerve regeneration and preservation in corneal transplantation and dry eye mouse models^22,23^.

So far, the in vivo studies that have tested the impact of these inhibitors on neuronal regrowth after injury have employed injury models that lead to changes in other cell types responding to tissue injury in the microenvironment around injured nerves. Because Semaphorin proteins play a role in inflammation, angiogenesis, and remyelination of neurons^18,24^, we undertook our current study to test the effects of Vinaxanthone, a described specific inhibitor of Sema3A, on isolated neurons, separating the effects of other cell types involved in neuronal growth. For this, we tested if Vinaxanthone (Vx) could block both the axonal growth cone collapse induced by Sema3A in embryonic neurons and the growth promoting effects induced by Sema3A in adult neurons. This approach allowed us to examine if this inhibitor acts directly on Sema3A activity in neurons or if its pro-regenerative effect seen in vivo may be related to additional targets involved in nerve regeneration.

## Materials and methods

### Animals

Work with animals on this study has been approved by the University of Illinois-Chicago, Institutional Animal Care and Use Committee. All experiments were performed according to the guidelines of the Association for Research in Vision and Ophthalmology Statement for the Use of Animals in Ophthalmic and Vision Research under the approved protocol 20-222 and in compliance with the Arrive guidelines. The C57BL/6 mouse strain was used to isolate embryonic dorsal root ganglia neurons and the neurofluorescent thy1-YFP mouse strain was used for isolation of trigeminal ganglia neurons. Both mouse strains were purchased from Jackson Laboratories (Bar Harbor, ME) and bred as described^25^. All mice were fed a standard diet ad libitum and maintained on a 12-h light–dark cycle.

### Vinaxanthone preparation

The natural product Vinaxanthone was synthetized in Dr. Dionicio Siegel’s laboratory (UC San Diego, Skaggs School of Pharmacy and Pharmaceutical Sciences). He kindly provided analytically pure Vinaxanthone for these studies. The reagent was suspended in DMSO and prepared as stock solution of 100 mg/mL and stored at − 80 °C until use. Vinaxanthone was diluted to appropriate concentration in culture medium right before addition to neurons. The reported in vitro IC50 for Vinaxanthone is approximately 0.09 µg/mL (0.16 µM)^21,26^. In our study we used a concentration range from 0.05 to 2 µg/mL to test its effect in adult trigeminal ganglia neurons since this has not been previously investigated.

### Isolation of embryonic dorsal root ganglia neurons

Time mating breeding pairs of C57Bl/6 mice were set up to isolate E15 embryonic dorsal root ganglia neurons as described^12^. Briefly, pregnant mice were sacrificed by CO_2_ inhalation followed by cervical dislocation, embryos were excised and decapitated on a cold HBSS solution. The vertebral column was dissected out from all other tissues and placed on a clean dish for dissection. The spinal cord and meninges were removed and axons were trimmed before extracting the DRG. The ganglia were placed in ice cold HBSS and transferred to a tube for enzymatic digestion with papain for 8 min at 37 °C followed by collagenase/dispase digestion for 8 min at 37 °C (Worthington Biochemicals, Lakewood, NJ). DRG neurons were centrifuged at 400*g* for 3 min, washed once with warm Neurobasal medium and plated on laminin/poly-d-lysine (Sigma) coated bottom glass 6 well plate (Cellvis cat number P06-20-1.5-N) and incubated for 2 h. The neurons were incubated with 50 ng/mL nerve growth factor (NGF, cat number N60009, Sigma) to induce neurite growth. If necessary, NGF was replenished every other day.

### Inhibition assay of embryonic dorsal root ganglion neurons

Embryonic neurons treated with NGF developed abundant neurites with clear axon growth cones 24 h after plating. Neurons were either left untreated to follow the normal growth of the neurites or the culture medium was replaced with fresh medium containing 0.1 μg/mL Vinaxanthone or 100 ng/mL Sema3A (Catalog number 1250-S3, R&D Systems, Minneapolis, MN). After 1 h incubation with Vinaxanthone, 100 ng/mL Sema3A was added to these wells and time lapse imaging was started to determine the effects of these treatments on axonal growth cones. Images were recorded every 20 min for a period of 6 h using an AxioObserver fluorescent microscope (Carl Zeiss Microimaging GmbH, Jena, Germany). Around 12–15 neurons were imaged and quantified per dish, treatments were performed in duplicate, so 24–30 neurons were evaluated per experiment. A total of four experiments were performed.

### Isolation of trigeminal ganglia neurons

Primary neurons from the trigeminal ganglia were obtained from 3 to 4 week-old Thy1-YFP mice and cultured as previously described^12,25^. In brief, mice were sacrificed by CO_2_ inhalation followed by cervical dislocation and the ophthalmic branches of the trigeminal ganglia were harvested and subjected to enzymatic digestion with papain followed by a collagenase/dispase mixture. Trigeminal neurons were separated in Percoll (Sigma, St. Louis, MO) gradients by centrifugation at 1300*g* for 10 min and seeded on poly-d-lysine coated glass bottom dishes (Cellvis Cat # D35-20-1.5-N, Mountain View, CA) containing Neurobasal A medium (Invitrogen, Carlsbad, CA) supplemented with 1% B27 supplement (Invitrogen, Carlsbad, CA) and 1% penicillin/streptomycin antibiotics (Gibco, Grand Island, NY). Isolated neurons were incubated and allowed to attach for 2 h before initiation of the treatments. For every experiment one dish of neurons was left untreated as a negative control, one dish was treated with NGF at 50 ng/mL as a positive control for neuronal growth induction or with 50 ng/mL Sema3A, while the rest were used for experimental conditions. The cultures were replenished with the same treatment growth media every other day.

### Neurite formation and elongation assay of adult trigeminal ganglion neurons

Neuronal growth was established as those neurons that presented neurite extension that was two-fold greater than the diameter of the cell body. Neurite elongation was classified into three types: (i) short (neurites between 50 and 100 μm in length, examples in Fig. 2A Vx 0.5 and Vx 1–2 treatments), (ii) medium (neurites between 150 and 400 μm in length, see Fig. 2A control and Vx 0.05–0.25), and (iii) long (neurites longer than 400 μm, see Fig. 2A NGF and Sema3A treatments), similarly to our previous publication^12^. All neurons in the dish were counted to obtain the percentage of neurons with neuronal growth. Experiments without neuronal growth in the NGF-positive control condition were excluded from analysis. Images were obtained using an AxioObserver fluorescent microscope and processed with Neuron J software (an Image J plugin available at https://imagescience.org/meijering/software/neuronj) was used to trace neuronal length and branching. Additionally, images were analyzed for neuronal length and branching using the Sholl analysis on Neurolucida software. For publication, all TG neuron fluorescent images were converted to grayscale mode and inverted to improve contrast using Photoshop 21.1.3 version.

### Effect of Vinaxanthone on adult neuronal growth

On the day of isolation (Day 0) neurons were left untreated or treated singly with 50 ng/mL NGF, 50 ng/mL Sema3A or Vinaxanthone in a dose response manner (0.05–2 μg/mL). Neuronal growth was evaluated on Day 3 and 4 post treatment and determined by counting all neurons on the dish and quantifying those with neurite outgrowth as described above. Neuronal length and branching were evaluated on all neurons that presented medium to long neurites as described above.

### Inhibition assays of adult trigeminal ganglion neurons

For Vinaxanthone inhibition studies we performed two different experiments to evaluate its effect. First, Vinaxanthone was added to the cultures before Sema3A or NGF to assess its role on the initiation of neurite growth. Secondly, Vinaxanthone was added after the neurons were treated with Sema3A or NGF to evaluate its effects on the progression of neurite growth. For the first set of experiments, on Day 0 TG neurons were incubated for one hour at 37 °C with Vinaxanthone in a dose response manner and then treated with either NGF or Sema3A at 50 ng/mL. Neuronal growth was evaluated at Day 2 and 3 (48 and 72 h) post seeding. For the second set of experiments, on Day 0 neurons were treated with 50 mg/mL NGF or Sema3A then incubated for 24 h to allow for neuronal growth to develop. The next day (Day 1) the culture media was replaced with either media alone, or media containing different doses of Vinaxanthone to test for inhibitory effects on the ongoing neurite sprouts. On Days 2 and 3 the neurite growth was assessed for all the treatments. For these experiments all neurons, including Thy1 positive and non-fluorescent, were counted, and images were obtained with an AxioObserver Z1 fluorescence microscope in the YFP or bright field channels attached to an AxioCam HRm digital camera (Zeiss) operated by Zen blue software and quantified as above.

### Immunostaining of trigeminal and dorsal root ganglia neurons

Trigeminal ganglia from 8–10 week old male and female C57Bl/6 mice were collected and treated with Sema3A at 50 ng/mL. After 3 days in culture, neurons were fixed with 4% PFA for 20 min and carefully washed 3 times with PBS for 5 min each on a slow speed orbital shaker. The dishes containing neurons were halfway filled with PBS and stored at 4 °C until processing for immunofluorescence staining. Dorsal root ganglia from E15 embryos were isolated and seeded on 12 well glass bottom plates. After 2 h incubation at 37 °C, neurons were treated with 50 ng/mL NGF. After 2 days in culture, neurons were fixed with PFA as for TG neurons described above. For immunofluorescence staining, neurons were incubated with blocking buffer (2% BSA, 2.5% donkey serum in PBS) for 1 h at room temperature and then incubated overnight with primary antibodies (see Table 1) at 4 °C in same blocking buffer. Neurons were washed 3 times in PBS for 5 min each and incubated at room temperature for 1 h with secondary antibody (see Table 1) in blocking buffer. Neurons were washed 3 times with PBS, kept in PBS and imaged at 20 × magnification using an AxioObserver fluorescent microscope (Carl Zeiss Microimaging GmbH, Jena, Germany) and processed with Adobe Photoshop using the same adjustment for all neurons. Neurons treated with secondary antibody alone were tested for background interference and this is shown on Fig. S4.

**Table 1:** Antibodies used for immunofluorescence staining of trigeminal and dorsal root ganglia neurons.

Primary antibody	Source	dilution	Vendor	Secondary antibody	dilution	Vendor
Neuropilin 1	Rabbit	1:200	Santa Cruz SC-5541	Donkey anti rabbit Alexa green 448	1:400	Jackson Immunoresearch 711-545-152
Neuropilin 2	Rabbit	1:200	Santa Cruz SC-5542	Donkey anti rabbit rhodamine	1:400	Jackson Immunoresearch 711-025-152
PlexinA1	Goat	1:400	R&D Systems AF4309	Donkey anti goat Alexa green 448	1:400	ThermoFisher A11055
PlexinA3	Rabbit	1:400	Santa Cruz SC-25641	Donkey anti goat TRITC	1:400	Jackson Immunoresearch 705-025-003
TrkA	Rabbit	1:200	Santa Cruz SC-118	Donkey anti rabbit Alexa green 448	1:400	Jackson Immunoresearch 711-545-152
TrkB	Rabbit	1:200	Bioss Bs-0174R	Donkey anti rabbit rhodamine	1:400	Jackson Immunoresearch 711-025-152
CGRP	Rabbit	1:200	Bioss Bs-791R	Donkey anti rabbit Alexa green 448	1:400	Jackson Immunoresearch 711-545-152

### Statistics

Data are presented as mean ± SEM. All data were analyzed using GraphPad Prism version 8. The significance of differences was evaluated using analysis of variance (ANOVA) followed by Dunnett’s multiple comparisons test. Data that failed the Shapiro–Wilk test for normality were analyzed using the nonparametric Kruskal–Wallis test, followed by Dunn’s multiple comparisons test. For any statistical test, P < 0.05 was considered statistically significant.

## Results

### Vinaxanthone inhibits Sema3A induced axonal growth cone collapse in embryonic neurons

**Figure 1:**
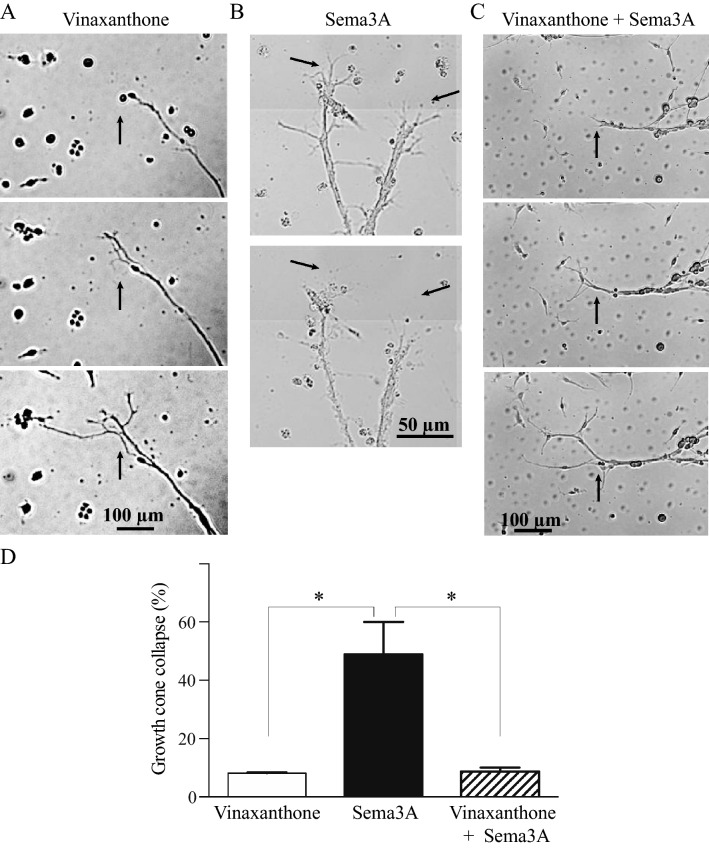
Vinaxanthone inhibits Sema3A-induced growth cone collapse in isolated embryonic DRG neurons. Isolated neurons from E15 wildtype mice were incubated with NGF to induce neurite sprouts. After 24 h abundant neurites with well-developed axon growth cones were observed. Addition of Vinaxanthone does not induce neurite regression or collapse of the axon growth cones (**A**), while Sema3A rapidly induces collapse of the axonal growth cones (**B**). Pre-incubation of the neurons with Vinaxanthone for 1 h before adding Sema3A blocked the axonal regression effect due to Sema3A and neuronal growth continued (**C**). The treatment’s effects on neuronal growth and regression were recorded via time lapse images (see Supplementary Videos S1, S2 and S3 in Supplementary Information) and analyzed as described in “Materials and methods”. The 50% increase in axon growth cone collapse induced by Sema3A, was reduced to basal levels in the presence of 0.1 μg/mL Vinaxanthone (**D**). Data represent the mean ± SEM of four independent experiments, with approximately 100 neurons were evaluated per treatment, a P value < 0.05 was considered statistically significant between the treatments, arrows indicate the starting point for video recording of neurite elongation/regression and selected time frames show the treatment effect.

In order to validate the activity of Vinaxanthone in our experiments, we tested the effect of this compound in a classical growth cone collapse assay in embryonic neurons^12^. Isolated neurons were treated with NGF, which induced potent neurite sprouting from the embryonic neurons. Representative images in Fig. 1 show the effects of the different treatments. Addition of Vinaxanthone does not change the neuronal growth induction (Fig. 1A). However, addition of Sema3A to the NGF-treated neurons induced neurite regression that started with growth cone collapse (Fig. 1B) and could continue to full neurite regression (data not shown). Pre-incubation of neurons with Vinaxanthone prior to the addition of Sema3A prevented axonal growth cone collapse, and neurites continued to grow as shown in Fig. 1C. Sema3A addition to NGF-treated neurons led to the collapse of approximately half of the observed growth cones (Fig. 1D). Vinaxanthone supplementation without Sema3A addition did not induce growth cone collapse (Fig. 1D).

### In the absence of growth factors, treatment with Vinaxanthone does not promote neurite formation or elongation in adult TG neurons

**Figure 2:**
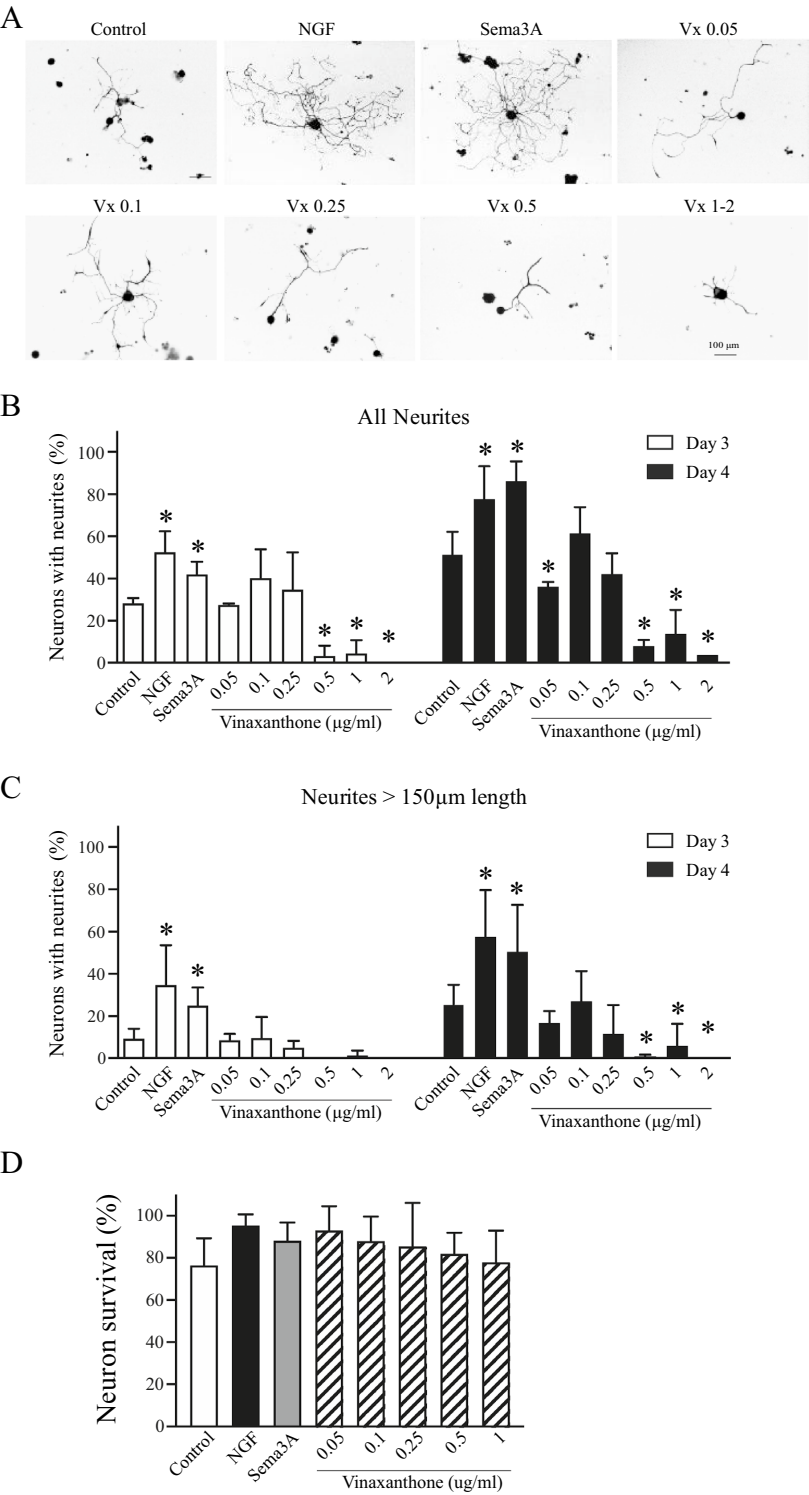
Effect of Vinaxanthone on neurite formation in adult TG neurons in the absence of growth factors. Lower doses of Vinaxanthone did not increase neurite formation while higher doses of Vinaxanthone inhibited neuronal growth. Isolated trigeminal neurons were left untreated or treated with either NGF or Sema3A at 50 ng/mL or with Vinaxanthone in a dose response manner. Neurite growth was analyzed after 3 and 4 days in culture by counting all neurons on the dish and tallying those that presented neurite outgrowth. (**A**) Representative images of neurite growth induced by the indicated treatments on TG neurons. (**B**) All neurons were counted for each treatment. NGF and Sema3A induced overall neuronal growth, while no growth promoting effects were seen in neurons treated with Vinaxanthone. Vinaxanthone concentrations under 0.25 μg/mL led to similar proportions of neurons exhibiting neurite outgrowth to control dishes. Vinaxanthone at a dose of 0.5 μg/mL or higher inhibited the sprouting of neurites. (**C**) The percentage of neurons with neurites over 150 μm in length was evaluated for each treatment. The effects of NGF, Sema3A and lower concentration Vinaxanthone on medium to large neurite sprouts were similar to those described above. However, longer neurites were more affected by Vinaxanthone at doses of 0.5 μg/mL or higher. (**D**) Neuronal survival was evaluated by counting all neurons on the dish from Day 3 to Day 4 of culture, no significant difference was observed among the treatments.

The effect of Vinaxanthone in adult TG neurons was evaluated in a dose response manner by assessing neuronal growth compared to the growth induced by growth medium alone (negative control) or media supplemented with NGF or Sema3A. All neurons in the dish were counted to obtain the percentage of neurons with neurite growth and determine the treatment effect on the proportion of neurons with neurites and on the elongation of the neurites. Single incubations with Sema3A, NGF, and Vx had differential effects on neurite formation by adult TG neurons (Fig. 2A). Both NGF and Sema3A induced neurite formation to a similar degree, which was significantly higher than the level seen in the control condition. Neurite formation after supplementation of Vinaxanthone in the absence of Sema3A or NGF was similar to the untreated control condition, except at concentrations of 0.5 μg/mL or higher. At these high concentrations there was a significant reduction in neurite formation on Day 3 of culture compared to NGF, Sema3A or untreated control. An evaluation of neurite formation on Day 4 of culture, revealed that NGF and Sema3A continued to promote increased neurite formation after the additional day of culture. At four of the tested concentrations, Vx led to a significant decrease in neurite formation as compared to the NGF, Sema3A or untreated control neurons (Fig. 2B).

To examine whether Vx has an effect on neurite elongation that is separate from the process of neurite initiation, we analyzed the impact of Vx treatment on length of elongated neurites (Fig. 2C). Neurons treated with NGF and Sema3A had significantly longer neurites on Day 3 compared to the control condition; this effect persisted after an additional day of culture. On Day 3 at any tested concentration, Vx failed to promote elongation compared to control. As compared to the untreated control, none of the tested concentrations of Vx significantly enhanced elongation after an additional day of culture, and Vx supplementation at 0.5 μg/mL or higher resulted in significantly shorter neurites by Day 4 of culture.

Data represent the mean ± SEM of three independent experiments and all neurons in a dish were analyzed (average 80 neurons per treatment/experiment), a P value < 0.05 was considered statistically significant between the treatments (*Statistically significant treatment vs control, all images at same magnification, scale bar = 100 μm).

To rule out the possible confounding effect of Vx toxicity that could explain the low percentage of neurons with neurites at high Vx concentrations, we compared the survival of neurons from Day 3 to Day 4 of culture among all tested conditions (Fig. 2D). We did not see a significant impact of Vx concentration on cell survival even at high concentrations of Vx, and the observed values were similar to control, NGF or Sema3A treated neurons. Overall, these results show that Vinaxanthone treatment by itself, in the absence of other growth factors, did not lead to increased neurite formation or elongation, and when used at its suggested IC50 of 0.1 μg/mL no significant differences were observed compared to control neurons. However, high concentrations of Vx may have a potentially inhibitory effect on neurite formation.

**Figure 3:**
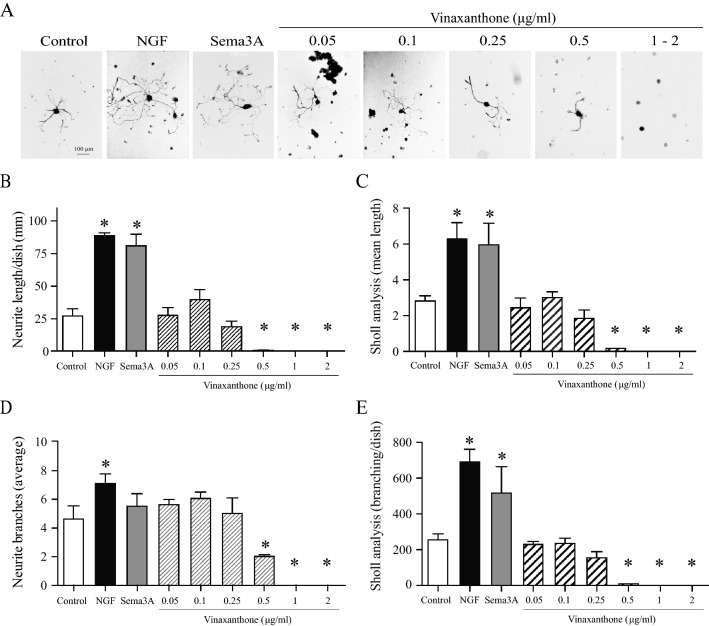
Effect of Vinaxanthone on neurite elongation and branching in adult TG neurons in the absence of growth factors. Isolated trigeminal neurons were left untreated or treated with either NGF or Sema3A at 50 ng/mL or with Vinaxanthone in a dose response manner. (**A**) Representative images of neurite elongation and branching observed after 3 days of culture with the indicated treatments. (**B**,**C**) Quantification of neurite length showed that NGF and Sema3A induced strong neurite outgrowth, while Vinaxanthone at a dose of 0.25 μg/mL or lower showed similar effects to control dishes. However, Vinaxanthone at a dose of 0.5 μg/mL or higher inhibited the formation of long neurites to a level below the untreated control condition. (**D**) Similar average neurite branching was observed among all treatments that presented elongated neurons. (**E**) However, Sholl analysis showed that NGF and Sema3A induced a significant increase in branching compared to all other conditions. (**D**,**E**) A reduction in branching was only observed in neurons treated with Vinaxanthone at 0.5 μg/mL or higher. At higher doses no branching was observed. Data represent the mean ± SEM of three independent experiments and images of 10–15 neurons/treatment were analyzed, P value < 0.05 was considered statistically significant between the treatments (*statistically significant treatment vs control, all images at same magnification, scale bar = 100 μm).

The quantification of neurite length and branching for all neurons with elongated neurites (> 150 μm length, Fig. 3) further highlights the lack of neurite induction by Vx. Figure 3A includes representative images of neurite elongation by Day 3 of culture and shows that only NGF and Sema3A treatments induced significant elongation. Quantification in Fig. 3B,C indicates that Sema3A and NGF induced a similar level of neurite elongation that was significantly greater than that seen in the control condition. All tested concentrations of Vx led to significantly shorter neurites as compared to NGF or Sema3A. Furthermore, concentrations of 0.5 μg/mL Vx or higher significantly decreased neurite lengths to levels below those achieved in the control condition. Thus, supplementation of dishes with Vinaxanthone at 0.5 μg/mL or above almost completely blocks neurite elongation. As axonal branching is a process that is imperative to proper neuronal connectivity and functionality, we evaluated if Vx treatments had an impact on branching (Fig. 3C,D). Vinaxanthone’s influence on neurite branching was similar to its influence on neurite formation and elongation. Vinaxanthone concentrations under 0.5 μg/mL did not affect neurite branching in those elongated neurons (Fig. 3D), but the total branching in Vx treated neurons was significantly reduced when compared to NGF or Sema3A treatments (Fig. 3E). Concentrations of 0.5 µg/mL Vx or above significantly decreased branching of neurons compared to NGF, Sema3A or the control.

### In the presence of growth factors, Vinaxanthone fails to inhibit neurite formation in adult TG neurons

**Figure 4:**
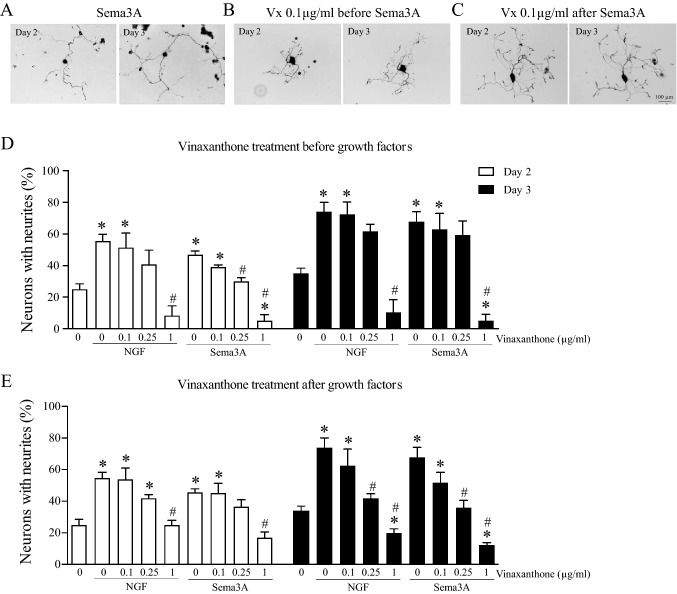
Vinaxanthone at its IC50 fails to inhibit the growth promoting effects of both Sema3A and NGF in adult TG neurons. Isolated TG neurons were left untreated or treated with Vinaxanthone in a concentration-dependent manner before or after incubation with NGF or Sema3A. Representative images of neurite growth induced by Sema3A (**A**) and the effects of 0.1 μg/mL Vinaxanthone added before (**B**) or after Sema3A (**C**), the images shown correspond to the same neurons imaged on Day 2 and Day 3 for each treatment, respectively. (**D**) The neurons were pre-treated with different doses of Vinaxanthone for 1 h. Then, NGF or Sema3A at 50 ng/mL were added to the dishes and neuronal growth was evaluated on Days 2 and 3 of culture. Vinaxanthone used at its IC50 of 0.1 μg/mL does not inhibit the Sema3A or NGF induced neurite growth. At the highest tested dose, Vx pre-treatment led to a level of neurite formation that was significantly below the level seen in either the NGF or Sema3A treated conditions. (**E**) Neurons were treated with 50 ng/mL NGF or Sema3A and allowed to grow for 1 day, at which point the medium was replaced with new medium containing different doses of Vinaxanthone. After additional 24 and 48 h of culture, neurons were evaluated for neuronal growth, and we found a similar trend for neurite formation as demonstrated for the Vx pre-treatment condition. Data represent the mean ± SEM of four independent experiments, a P value < 0.05 was considered statistically significant between the treatments (^#^statistically significant treatment vs NGF or Sema3A alone, respectively; *statistically significant treatment vs control), all images at same magnification, scale bar = 100 μm.

The effect of Vinaxanthone on neurite growth induced by NGF or Sema3A was tested by adding the inhibitor before or after incubation of the TG neurons with these growth promoting agents. Figure 4A–C shows representative images of TG neurons’ responses to the Sema3A treatment alone or in combination with Vinaxanthone used at its IC50 of 0.1 μg/mL. Images show the neurite growth of the same neuron from Day 2 to Day 3. Sema3A-induced neurite growth was not inhibited by Vinaxanthone, regardless of whether the inhibitor was applied to the culture before or after Sema3A. Quantification of the neurite growth on Day 2 and Day 3 of culture is shown in Fig. 4D,E. Vinaxanthone pre-treatment (Fig. 4D), proceeded by the addition of NGF and Sema3A, does not inhibit NGF or Sema3A-induced neurite formation when used at 0.1 μg/mL. By Day 3 of culture, the proportions of neurons with neurites in the 0.1–0.25 μg/mL Vinaxanthone conditions were generally not significantly different from the singly-treated NGF or Sema3A conditions and usually resulted in a level of neurite formation that was also not statistically different from the negative control. However, significant inhibition was observed both for Sema3A and NGF treated neurons (Fig. 4D) at the highest tested concentration of Vx, (1 μg/mL) on Day 2. These patterns persisted after an additional day of cell culture. Additionally, the neuronal growth induced by different doses of Sema3A (15–50 ng/mL) was not inhibited by Vinaxanthone used at its IC50 (Fig. S1).

In order to test whether Vx supplementation has a modulatory role on neurites that are already established, as would be the case within in vivo models of axonal injury, we tested the impact of Vx supplementation on neurons that have been pre-treated with either NGF or Sema3A (Fig. 4E). Neurons treated with either NGF or Sema3A without Vx supplementation showed increased proportions of neurons with neurites as compared to the untreated control (Fig. 4E). Similarly, neurons treated with 0.1 μg/mL Vx showed significantly higher neurite outgrowth when compared to untreated neurons but same levels when compared to NGF or Sema3A treatments. When neurons received Vx at doses above its IC50, we observed a mixed response on Day 2. However, at Day 3 it was evident that Vinaxanthone at higher doses of 1 μg/mL inhibited neurite growth when compared to NGF, Sema3A or untreated control. Whereas at an intermediate Vx dose of 0.25 μg/mL the percentages of neurons with neurites were significantly different compared to those observed for NGF or Sema3A, but on the same level of untreated control. The combined results from Fig. 4 demonstrate that Vx at its IC50 of 0.1 μg/mL does not significantly inhibit neurite formation induced by either NGF or Sema3A and has a non-specific inhibitory role on neurite formation when used at high concentrations.

### TG and DRG neurons express Sema3A, NGF and BDNF receptors

**Figure 5:**
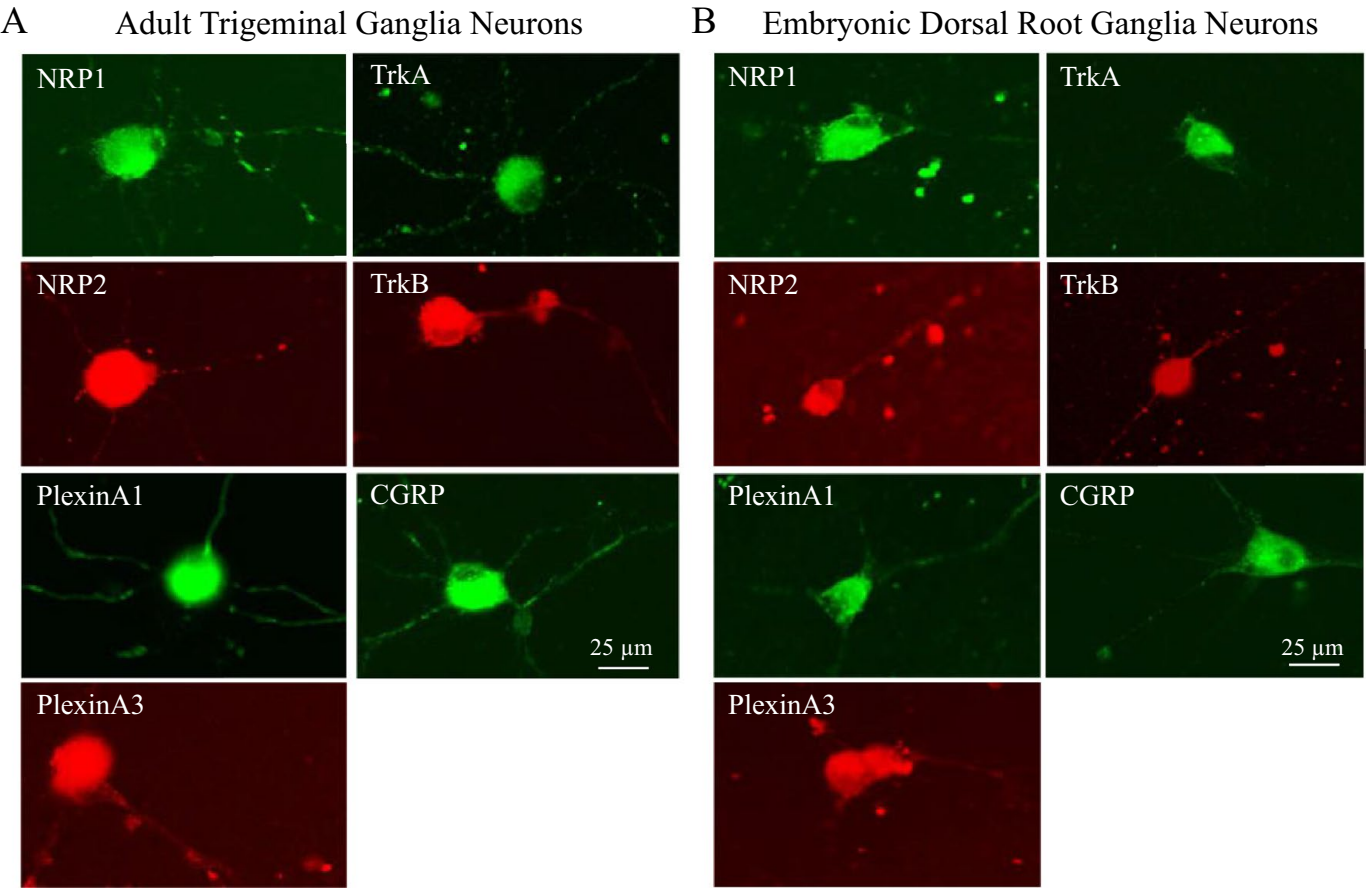
Expression of Sema3A, NGF and BNDF receptors in adult TG and embryonic DRG neurons. The expression of Sema3A receptors (NRP1, NRP2, PlexinA1 and PlexinA3) and the NGF receptors TrkA, the BDNF receptor TrkB, and the neuropeptide CGRP in isolated TG and DRG neurons was evaluated by immunofluorescence staining. Representative images of TG neurons treated with Sema3A (**A**) show expression of all receptors both in the cell body as well as in the growing neurites. We found that 100% of neurons express the receptors and the neuropeptide CGRP. Similarly, all of these receptors were also expressed in DRG neurons treated with NGF (**B**). However, 100% of the neurons expressed NRP1, NRP2, PlexinA3, TrkA and CGRP, while 75% of the DRG neurons expressed PlexinA3 and TrkB. In general, there were no major differences in Sema3A receptor expression between the adult and embryonic sensory neurons. Additionally, no differences were observed in the expression of the neuropeptide CGRP. See Table 1 in “Materials and methods” for antibody information. All images were taken at same magnification, scale bar = 25 μm. The background effect generated by the secondary antibody (staining without primary antibody) is shown on Supplementary Fig. S4).

We attempted to evaluate if the differential effects of Vinaxanthone seen in embryonic DRG versus adult TG neurons could be explained by differential expression of receptors that interact with Sema3A. We performed immunofluorescence staining on adult TG neurons treated with Sema3A and compared it to that of embryonic DRG neurons treated with NGF. We evaluated the expression of the Sema3A receptors NRP1, NRP2, Plexins A1 and A3, and found no major differences in the expression between adult or embryonic neurons. Similarly, the expression of the NGF receptors TrkA, the BNDF receptor TrkB and the neuropeptide CGRP was comparable in both neuronal types. Similarly, no obvious differences were observed in the expression both in the soma or neurite extension (Fig. 5). We found that only 75% of the DRG neurons expressed PlexinA1 and TrkB, while 100% of TG neurons express these receptors. All other tested receptors and CGRP were expressed in 100% of both TG and DRG neurons.

## Discussion

In this study we demonstrate that Vinaxanthone promotes axonal growth cone integrity via its anti-Sema3A activity in embryonic PNS neurons but fails to inhibit Sema3A induced neurite formation in adult primary neurons. When applied singly and at its most commonly used concentration in vitro (0.1 μg/mL), Vinaxanthone did not significantly influence neurite formation in adult trigeminal ganglion neurons. However, at the highest tested concentrations Vinaxanthone prohibited formation of neurites to levels below those seen in the untreated conditions.

Vinaxanthone is currently being evaluated as a potential pro-neural therapeutic for injuries, because it has been described as an inhibitor of Semaphorin3A, a factor classically thought to prevent neuronal regeneration. Prior studies on the peripheral nervous system have assessed Vinaxanthone’s IC50 to be 0.1–0.2 μM, evaluated by the axonal growth cone collapse assay on DRG neurons isolated from embryonic chicks or rats^21,26,27^. As expected from these previous works, in our embryonic DRG culture Vinaxanthone used at its IC50 of 0.1 μg/mL prevents the collapse of growth cones caused by Sema3A stimulation. However, we find that when tested on adult primary neurons, supplementation with Vinaxanthone fails to inhibit the Sema3A induced neuronal growth even when Sema3A was used at lower doses, well below its most potent growth promoting effects (Fig. S1). Additionally, we found that Vinaxanthone started presenting off target effects when used at higher doses. In neurons treated with a combination of NGF or Sema3A and Vx, high doses of Vx had a partial inhibitory effect on neurite formation, irrespective of whether Vx was added prior to or post incubation with the growth factors. At the highest Vx concentration tested in the present study, the small molecule inhibitor prevented neurite outgrowth, even when the neurons were treated with NGF. To our knowledge, there have been no studies linking NGF and Vinaxanthone, thus this interaction may point to an effect of Vx on neurite formation that is not specific to Sema3A signaling or that might be shared by both NGF and Sema3A signaling cascades. Moreover, instead of acting as a pro-regenerative molecule for adult neurons, Vinaxanthone has an anti-regenerative effect at the highest concentrations studied in our model.

There are a number of possible explanations for the seemingly contradictory findings regarding the inhibitory effects of Vinaxanthone on Sema3A activities. First, we acknowledge that mixed conclusions have been reached on studies investigating the effect of Sema3A on adult neurons^28^, although the majority of studies consider Sema3A to be a non-permissive cue to regeneration and, therefore, its inhibition would be pro-regenerative. For example, a study by Chin et al. showed that Vinaxanthone induced neuronal regeneration of transected neurons in *C*. *elegans* of the larval (not adult) stage^29^. However, several studies, including our own, indicate the growth-promoting effects of Semaphorins on adult neurons, and currently Sema3A, 3B, 3C, 3F, and 7A, have all been demonstrated to induce neuronal growth in vitro and in vivo^11–14,30–33^. A level of specificity in signaling among the semaphorins is conferred by the combinations of Neuropilin, Plexin, and other co-receptors within the receptor binding complex. Sema3B binds Neuropilin-2 and Plexin-A1, -A2 or -A4^34^. Our immunostaining shows that TG and DRG neurons express the Sema3A receptors NRP1, NRP2, PlexinA1 and PlexinA3. It is known that Vinaxanthone interferes with the binding of Sema3A to NRP1 on embryonic neurons. However, our preliminary studies demonstrate that the growth promoting effects of Sema3A for adult neurons may be independent of its interaction with NRP1. We showed that a neutralizing antibody against Neruropilin-1 failed to inhibit the Sema3A growth promoting effect in adult TG neurons while inhibiting the effect of VEGF A, a neurite growth-promoting molecule that relies on NRP1 as a co-receptor. Mice deficient in PlexinA3 and PlexinA4, two key receptors for class 3 Semaphorins, and which received spinal cord transection, did not show enhanced regeneration, suggesting that inhibiting Semaphorin signaling may not be sufficient for enhanced nerve regeneration^35^. Future work is required to determine the differences, if any, of the membrane bound receptors and co-receptors that interact with Sema3A in developing versus postnatal neurons. This difference, if any, may explain the observed dissimilar effects of Vx in embryonic and adult neurons. It is also possible that intracellular signals may be responsible for our observed results. A potential intracellular mechanism that regulates the dual repulsive and attractive roles of Sema3B within the anterior commissure is known. This attractive effect is achieved due to the recruitment of Focal Adhesion Kinase (FAK) and src family kinases, both of which are known to mediate the chemoattractive function of netrin, a classical attractive cue during development^33^.

Attractive effects of Sema3A on neurons have also been reported within the CNS. Sema3A has a well-known duality of function in the case of cortical pyramidal neurons, as it acts as a chemoattractant for dendritic growth and a chemorepellent for axonal growth of the same neuron, thus helping to pattern cortical layers^36^. Recently, an in vivo study achieved regeneration of cortical tissue using Sema3A gradients. Adult rats with frontal lobe injury that received a hydrogel implant loaded with a gradient of Semaphorin3A demonstrated increased neural progenitor migration to the injured area and subsequent differentiation into cortical tissue with a similar transcriptomic signature to uninjured brains^37^. Some aspects of the mechanisms behind this duality of Sema3A function have been elucidated and involved several signals pathways such as integrin dependent phosphorylation of Focal Adhesion Kinase, extrinsic supplementation of cGMP, and activation of the L1 Cellular Adhesion Molecule^38–40^.

Beyond the possible differences in Semaphorin-receptor binding and intracellular mediators during development and adulthood, there is also an alternative explanation of our findings related to the non-specific binding of Vinaxanthone and Xanthofulvin to other cellular targets. Xanthofulvin was originally discovered as an antifungal agent by blocking chitin synthase and has been tied to inhibition of a number of mammalian proteins involved in neuroregenerative or wound healing processes, including Epidermal Growth Factor Receptor, Ephrin A4, Fibroblast Growth Factor Receptor 3, and Matrix Metalloproteinase 2^21,41–46^. Vinaxanthone has inhibitory activity on anti-Phospholipase C, the CD4 helper T cell receptor, and bacterial fatty synthesis enzyme FabI^16–18,47^. Recently, a study proposed that Vinaxanthone and Xanthofulvin are positive allosteric modulators of the succinate receptor 1, a G protein coupled receptor (GPCR) that is involved in the pro-angiogenic response pathway in a variety of tissues^20^.

In the present study, higher doses of Vx have a partial inhibitory effect on neurite formation induced by Sema3A or NGF. Vinaxanthone has not been previously described to interact with NGF or its main receptors TrkA or p75. Our immunostaining demonstrates that both DRG and TG neurons express the receptors for Sema3A, NGF and BDNF. Additionally, our studies in adult TG neurons show that they respond equally to both NGF and Sema3A, and the combination of both factors does not induce significant changes in the percentage of neurons that have neurites, nor their length, branching or complexity (Fig. S2). Since the effects of NGF and Sema3A in promoting neurite growth are clearly not additive, it suggests that Sema3A promotes neurite growth predominantly from NGF-responsive adult neurons, therefore they are acting on the same neuronal population. An overlap between Sema3A and NGF signaling has been described previously^48,49^. Vinaxanthone’s partial inhibition of neurite growth in the presence of Sema3A or NGF hints that this inhibitory effect, especially when tested at high Vx concentrations, may be due to off-target effects of Vx that interfere with the general neuritogenesis pathways and is not specific to Sema3A signaling.

In vivo studies that have tested the effect of Vinaxanthone or other Sema3A inhibitors on neuronal regeneration in the eye have utilized a number of models of nerve injury. Omoto et al. evaluated the effects of Vinaxanthone subconjunctival injections on regeneration of corneal nerves after 2 mm diameter corneal transplantation in mice^22^. They determined that on week 3 post transplantation, injections of Vinaxanthone (0.1 mg/mL) led to better mechanical sensation and greater axonal growth area. Yamazaki et al. tested the effect of Vinaxanthone applied as a topical agent in a corneal nerve injury model that mimics severe murine dry eye disease due to the excision of the extra-orbital lacrimal glands^23^. After 4 weeks of treatment, corneal sensitivity and nerve density were both increased in this injury model when treated with 0.5 mg/mL Vinaxanthone subconjunctival injections.

The pro-regenerative effects of Vinaxanthone and other anti-Sema3A inhibitors in the in vivo studies could be explained by the molecules’ inhibition of Sema3A activity in non-neural cells that are known to play important roles in the recovery of nervous system from injury. The prior mentioned in vivo studies in the eye used corneal injury models, like lamellar keratectomy^15^, corneal transplantation^22^, and severe dry eye induction via lacrimal gland excision^23^. These models inflict extensive neuronal and surrounding tissue damage and have effects on the cornea beyond the epithelium, thus may pose a far greater risk for corneal fibrosis, neovascularization, and immune cell infiltration^50–52^. Due to the pleiotropic effect of Sema3A on various non-neuronal cell types, it is possible that the nerve regeneration-promoting effect of Sema3A inhibition in vivo is majorly impacted by the extraneural roles of Sema3A signaling post injury. Future work will be necessary to tease out the direct neural and indirect or extraneural effects of Sema3A stimulation and inhibition in vivo*.*

In conclusion, our study draws a distinction between the activity of Vinaxanthone in adult and embryonic PNS neurons. We found that Vinaxanthone promotes growth cone survival in the presence of Sema3A in embryonic neurons. However, it fails to inhibit Sema3A induced neurite formation in adult neurons and, in fact, at high concentrations inhibits the growth promoting effects of Sema3A or NGF. Future studies that aim to inhibit Sema3A signaling to promote adult neuronal regeneration should consider the holistic effects of Sema3A inhibition in neurons and other cell types responding to injury.

## Supplementary Information

Supplementary Information.

Supplementary Video S1.

Supplementary Video S2.

Supplementary Video S3.
