# Supplementary material for: Vinaxanthone inhibits Semaphorin3A induced axonal growth cone collapse in embryonic neurons but fails to block its growth promoting effects on adult neurons
Source: Sci Rep. 2021 Jun 21;11:13019. doi: 10.1038/s41598-021-92375-w (PMC8217491; doi:10.1038/s41598-021-92375-w)
Supplement: Supplementary file 1 — Supplementary Information. [file 41598_2021_92375_MOESM1_ESM.docx]

***Vinaxanthone inhibits Semaphorin3A induced axonal growth cone collapse in embryonic***

***neurons but fails to block its growth promoting effects on adult neurons.***

**Supplementary Information**

**Materials and Methods for supplementary information**

**Inhibition assays of TG neurons treated with different doses of Sema3A**

In these Vinaxanthone inhibition studies TG neurons isolated from 4 week old mice were seeded on glass bottom dishes and treated with 0.1 μg/ml vinaxanthone for 1 hour. Similar number of dishes were left untreated for the same incubation time. Then 0, 5, 15, 30 and 50 ng/ml of Sema3A was added to both untreated or Vx treated neurons and incubated at 37^o^C. Three days later the neuronal growth was assessed for all the treatments. The proportion of neurons with neurites was plotted for two independent experiments.

**Evaluation of neuronal growth induced by NGF and Sema3A alone or in combination**

TG neurons isolated from 4 week old Thy-1 YFP mice were seeded and treated with 50 ng/ml NGF, Sema3A or both. Untreated control received incubation medium only. After 3 days in culture neuronal growth was evaluated by assessing the number of neurons presenting neurites. For this, all neurons, Thy1 positive and non-fluorescent, were counted, and images were obtained with an AxioObserver Z1 fluorescence microscope in the YFP or bright field channel attached to an AxioCam HRm digital camera (Zeiss) operated by Zen blue software. Length, branching, and complexity of neurites, were evaluated by Sholl analysis using Neurolucida software.

**Effect of anti neuropilin-1 neutralizing antibody on Sema3A induced neuronal growth**

TG neurons from 4 week old C57 mice were seeded and incubated either with medium alone or medium containing 3 μg/ml of anti neuropilin-1 antibody (R&D systems, AF566) or 0.1 μg/ml Vinaxanthone. After 1h incubation, neurons were treated with 50 ng/ml NGF, 30 and 50 ng/ml Sema3A, or 30 ng/ml recombinant human VEGFA (R&D Systems, 293-VE/CF). After 3 days in culture, neurons were evaluated for neuronal growth by assessing the number of neurons presenting neurites over the total neurons.

**Supplementary video S1. Representative time lapse video showing the effect of vinaxanthone on neuronal growth.** This video provides images used for Fig 1A and shows the neurite growth observed when embryonic DRG neurons treated with NGF received addition of Vinaxanthone at 0.1 μg/ml.

**Supplementary video S2. Representative time lapse video of axonal growth cone collapse induced by Sema3A.** This video provides images used for Fig 1B and shows the early axonal growth cone collapse and regression that occurs when embryonic DRG neurons treated with NGF received the addition of Sema3A.

**Supplementary video S3. Representative time lapse video of axonal growth and neurite extension.** This video provides images used for Fig 1C and shows the neurite growth observed when embryonic neurons were treated with NGF followed by pre-incubation of neurons with Vinaxanthone prior to the addition of Sema3A.

**Supplementary Figure 1. The dose-dependent, growth promoting effect of Sema3A in adult TG neurons is not inhibited by Vinaxanthone.** Adult TG neurons were treated with medium alone or medium containing 5, 15, 30 or 50 ng/ml Sema3A. After 2 days of incubation, neurons were treated with 0.1μg/ml Vinaxanthone or medium alone. The next day neurons were counted and imaged for neuronal growth analysis. This figure shows the number of neurons that present neurites at day 3. Sema3A induced significant neurite growth when compared to controls. However, we did not find any significant differences between the treatments that received Vinaxanthone and those that did not, either for control or Sema3A treated neurons. This data demonstrates the Vinaxanthone used at its IC50, does not inhibit Sema3A induced neuronal growth in adult TG neurons even when Sema3A was used at doses below its more potent growth promoting effects. Data represent the mean ± SEM of two independent experiments, statistical analysis was performed to compare the differences between the neurons that received Vx versus those that did not, a p value < 0.05 was considered statistically significant between the treatments. (* = statistically significant vs control, # = statistically significant vs control + Vx, n.s. = not significant). Vx=Vinaxanthone.

**Supplementary Figure 2. The growth promoting effect of Sema3A in TG neurons is comparable to NGF alone or combination of Sema3A + NGF.** Adult TG neurons were treated with medium alone or medium containing 50 ng/ml NGF, 50 ng/ml Sema3A or 50 ng/ml Sem3A + 50ng/ml NGF. After 3 days of incubation, neurons were evaluated for neuronal growth by counting all neurons in the dish as well as those that presented neurite growth. Neurons were imaged to determine elongation and branching by using Neurolucida software to trace the neurons and perform Sholl analysis. We did not find any significant differences between the treatment that received NGF or Sema3A alone or in combination. While all the treatments that received these factors were significantly different from untreated controls, induction, elongation and branching of neurites were similar when compared amongst the treatments. This data suggests that Sema3A promotes neurite growth predominantly from NGF-responsive TG neurons. This can be concluded because the effects of NGF and Sema3A in promoting neurite growth are not additive, suggesting that they are acting on the same neuronal population. Data represent the mean ± SEM of two independent experiments and images of 30 neurons/treatment were analyzed, p value < 0.05 was considered statistically significant between the treatments (* = statistically significant treatment vs control).

**Supplementary Figure 3. Effect of anti neuropilin-1 neutralizing antibody on Sema3A induced neuronal growth**. Adult TG neurons were treated with medium alone, medium containing 3 μg/ml anti neuropilin-1 antibody or 0.1 μg/ml Vinaxanthone. Neurons were incubated for 1 hour and then treated with medium alone or medium containing 50 ng/ml NGF, 30 ng/ml Sema3A or 30 50 ng/ml Sem3A. After 3 days of incubation, neurons were evaluated for neuronal growth by counting all neurons in the dish as well as those that presented neurite growth. We found that the anti neuropilin-1 antibody at this concentration reduced the neurite formation on the VEGFA treated neurons by 50%. However, no effect was seen on NGF or Sema3A treated neurons. Similarly, addition of Vinaxanthone did not inhibit the Sema3A induced neuronal growth. This data indicate that the Sema3A induced neurite growth may not require the participation of the neuroplin-1 receptor. This observation needs further studies to determine the mechanisms for Sema3A induction of neuronal growth on adult TG neurons. Data represent the mean ± SEM of two independent experiments, a P value < 0.05 was considered statistically significant between the treatments. * = statistically significant treatment vs control, # = statistically significant vs VEGFA treatment.

**Supplementary Figure 4. Immunofluorescent staining of neurons with and without the first antibody**. Isolated neurons were stained as described in Material and Methods. Controls without the primary antibody (left panels) show the minimal background generated by using the secondary antibody only. Right panel shows examples of neurons stained with primary antibodies and their respective secondary antibodies as described in Table 1. Similar data was obtained for both TG and DRG neurons. Images are shown uncropped and similar adjustments in Photoshop were performed on all images. Scale bar = 50μm.
